# IgE sensitization in a cohort of adolescents in southern Sweden and its relation to allergic symptoms

**DOI:** 10.1186/s12948-019-0110-6

**Published:** 2019-04-02

**Authors:** Therese Sterner, Ada Uldahl, Åke Svensson, Magnus P. Borres, Sigrid Sjölander, Alf Tunsäter, Jonas Björk, Cecilia Svedman, Magnus Bruze, Laura von Kobyletzki, Hampus Kiotseridis

**Affiliations:** 1 Department of Dermatology, Skåne University Hospital, Jan Waldenströmsgata 16, 205 02 Malmö, Sweden; 2 Department of Clinical Sciences Malmö, Faculty of Medicine, Lund University, Malmö, Sweden; 3 Competence Center of Allergy, Asthma and COPD, Skåne Regional Council, Lund, Sweden; 4 Department of Maternal and Child Health, Uppsala University, Uppsala, Sweden; 5 Thermo Fischer Scientific, Uppsala, Sweden; 6 Division of Occupational and Environmental Medicine, Lund University, Lund, Sweden; 7 Department of Occupational and Environmental Dermatology, Skåne University Hospital, Malmö, Sweden; 8 Department of Respiratory Medicine and Allergology, Skåne University Hospital, Lund University, Lund, Sweden

**Keywords:** Adolescent, Allergy, Allergen components, Asthma

## Abstract

**Background:**

There is a strong and consistent association between IgE sensitization and allergy, wheeze, eczema and food hypersensitivity. These conditions are also found in non-sensitized humans, and sensitization is found among individuals without allergy-related diseases. The aim of this study was to analyse the sensitization profile in a representative sample of the population, and to relate patterns of allergens and allergen components to allergic symptoms.

**Methods:**

A population of 195 adolescents took part in this clinical study, which included a self-reported questionnaire and in vitro IgE testing.

**Results:**

Sensitization to airborne allergens was significantly more common than sensitization to food allergens, 43% vs. 14%, respectively. IgE response was significantly higher in airborne allergens among adolescents with rhinitis (p < 0.001) and eczema (p < 0.01). Among 53 children with allergic symptoms according to the questionnaire, 60% were sensitized. Sensitization to food allergens was found among those with rhinitis, but only to PR-10 proteins. None of the participants had IgE to seed storage proteins.

**Conclusion:**

The adolescents in this study, taken from a normal Swedish population, were mainly sensitized to grass pollen and rarely to specific food allergens. The major grass pollen allergen Phl p 1 was the main sensitizer, followed by Cyn d 1 and Phl p 2. Sixty-one percent reporting any allergic symptom were sensitized, and the allergen components associated with wheeze and rhinoconjunctivitis were Fel d 4, Der f 2 and Can f 5.

## Background

An individual’s sensitization profile is clinically important due to the associated risk of the development and persistence of asthma and allergy symptoms throughout life [1, 2]. The calculation and interpretation of the sensitivity and specificity of IgE tests require clinical data from the subject’s case history to back up the clinical diagnosis. A population-based cohort study on adolescents in southern Sweden recently reported on the prevalence of various allergic symptoms: wheeze 10%, rhinoconjunctivitis 13%, food hypersensitivity 12% and eczema 11% [3]. The same study also showed that a parental history of asthma, hay fever and eczema was significantly more common among children with allergic diseases, and that wheeze was more common among those with parents who smoked. Only a limited amount of data is available on IgE sensitization and allergic multi-morbidity among children at the population level [4–7]. Most studies have focused on allergy morbidity in younger children, not on the clinical evaluation of the diagnostic sensitivity and specificity among teenagers [8, 9]. The multi-morbidity of eczema, rhinitis and asthma is common but does not always depend on IgE sensitization [7, 10], however, at the preclinical stage, the IgE test facilitates the prediction of allergic rhinitis [5, 11]. Although there is a strong association between IgE sensitization and eczema, asthma and rhinitis, these diseases are also found in non-sensitized individuals [12], and IgE sensitization is also seen in individuals without allergy-related diseases [8, 13].

The aim of this study was to analyse the sensitization profile in a representative sample from a population-based cohort of adolescents in southern Sweden, and to relate allergens and allergen components to allergic diseases.

## Material and method

### Study population

**Fig. 1:**
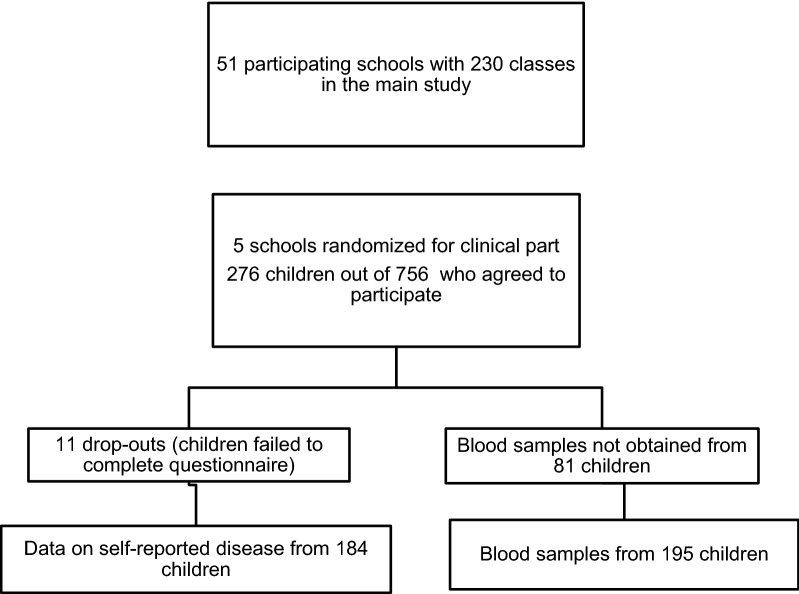
Flow chart

A random sample of 195 adolescents aged 13–15 years, was extracted from a larger population-based cohort in southern Sweden, described previously [3]. In 2012, 51 schools (230 classes) agreed to participate in the main cohort, from which a random sample was selected in the following way; The 51 participating schools in the main cohort were listed per municipality. A random sample table was used to select five schools. The heads of these five schools were contacted to obtain permission to visit the school for clinical examination of the participants and to collect blood samples. This resulted in a subpopulation of 756 adolescents from the five schools and of these, 276 adolescents got permission from their parent to participate in the study. Of these, 195 were at school by the time for the study examination. The participants were asked to complete a questionnaire, give blood samples and epicutaneous testing with a modified baseline series and a clinical skin examination were carried out by a dermatologist. The participants’ parents or guardians were also asked to complete a questionnaire. The flow chart is presented in (Fig. 1).

### Questionnaires

Two web-based questionnaires were used in this study, comprising 110 questions to the participants and 52 questions to the parents/guardian. The International Study of Asthma and Allergies in Childhood (ISAAC) questionnaire respiratory symptoms was completed by the participants [14–16]. The ISAAC questionnaire was designed as a tool for epidemiological research in asthma and allergic diseases. The questionnaire has been translated from English to Swedish according to the strict rules set up by a steering committee of ISAAC [16]. Questions were added about food allergy and/or intolerance, and dermatological symptoms such as contact allergy and eczema. The parental questionnaire included questions on the children; medication, home environment, habits, physical exercise and parental smoking . The location of the school was used as a proxy for the living area, since most children live close to their school in Sweden. The web-based questionnaires were constructed and provided by Clinical Studies Sweden, Forum South, Skåne University Hospital. Prevalences of wheeze, rhinoconjunctivitis, eczema and food hypersensitivity were obtained from the self-reported data in the questionnaire using the definitions given in Table 1.

**Table 1:** Definition of allergic symptom

Health condition	Definition
*Wheeze*	If the child answered yes to wheeze, at any time in their life and during the past year
*Rhinoconjunctivitis*	If the child answered yes to rhinitis (sneezing, runny or blocked nose without a cold) and conjunctivitis (itchy and/or watery eyes)at any time in their life and during the past year
*Eczema*	If the child answered yes to itching, rash/eczema for six months at any time during their life, and during the past year
*Food hypersensitivity*	If the child answered yes to a reaction to food at any time in their life and during the past year

Of the 195 study subjects, 36% reported wheeze, eczema, rhinoconjunctivitis or food hypersensitivity, while the other 64% did not report any of these symptoms as defined in Table 1. Among those with allergic symptoms, 47 had one symptom, 15 had two symptoms and 5 had 3 symptoms. None had all four symptoms. Food hypersensitivity was the most common reported symptom 16% (n = 30), followed by rhinoconjunctivitis 15% (n = 28), wheeze 10% (n = 18) and eczema 9% (n = 16). The most frequent combination of symptom was rhinoconjunctivitis and food allergy 3% (n = 5). *Demographic description*; 53% of the children were girls, allergic diseases among parents were more common among the 67 children with any of the allergic symptoms than among children without any allergic symptom.

### Serological analysis

Blood samples were collected during the period January–March 2013, and were stored frozen until analysis. Serum from 194/195 children was used for serological analysis. Specific IgE testing (s-IgE) was performed with two allergen panel tests: With Phadiatop™ Europe, fx5^™^ (Thermo Fisher Scientific, Uppsala Sweden; Table 2), sensitization was defined as an allergen-specific IgE level ≥ 0.35 kU/ I.

**Table 2:** Allergens analysed with Specific IgE testing

Phadiatop Common name of extract	Fx5 Common name of extract
House dust mite	Egg white
House dust mite	Atlantic cod
Cat dander	Wheat
Horse dander	Peanuts
Dog dander	Soybean
Timothy-grass	
Mould	
Birch	
Olive	
Mugwort	
Wall pellitory	

Serum was also tested for IgE reactivity to 112 allergen components (ISAC 112, Thermo Fisher Scientific, Copenhagen, Denmark). A positive response to a component was defined as IgE binding ≥ 0.3 ISU/l, and further classified into semi-quantitative categories; low (0.3–3 ISU), moderate (3–15 ISU) and high (15–150 ISU) [17].

### Statistical analysis

All statistical analysis was performed using the SAS^®^ statistical software system version 9.3 (SAS Institute Inc., Cary US) and R version 3.2.3 (R Foundation for Statistical Computing, Vienna, Austria). All statistical tests were two-sided and a significance level of < 0.05 was regarded as statistically significant. Fischer’s exact test was used to compare prevalences across groups.

Cluster analysis was performed on the IgE binding categories for all samples. Clustering was performed using sparse hierarchical clustering with complete linkage, as implemented in the R package *sparcl*, where the tuning parameter controlling the number of features was determined as the value providing the largest Gap statistic [18].

## Results

**Fig. 2:**
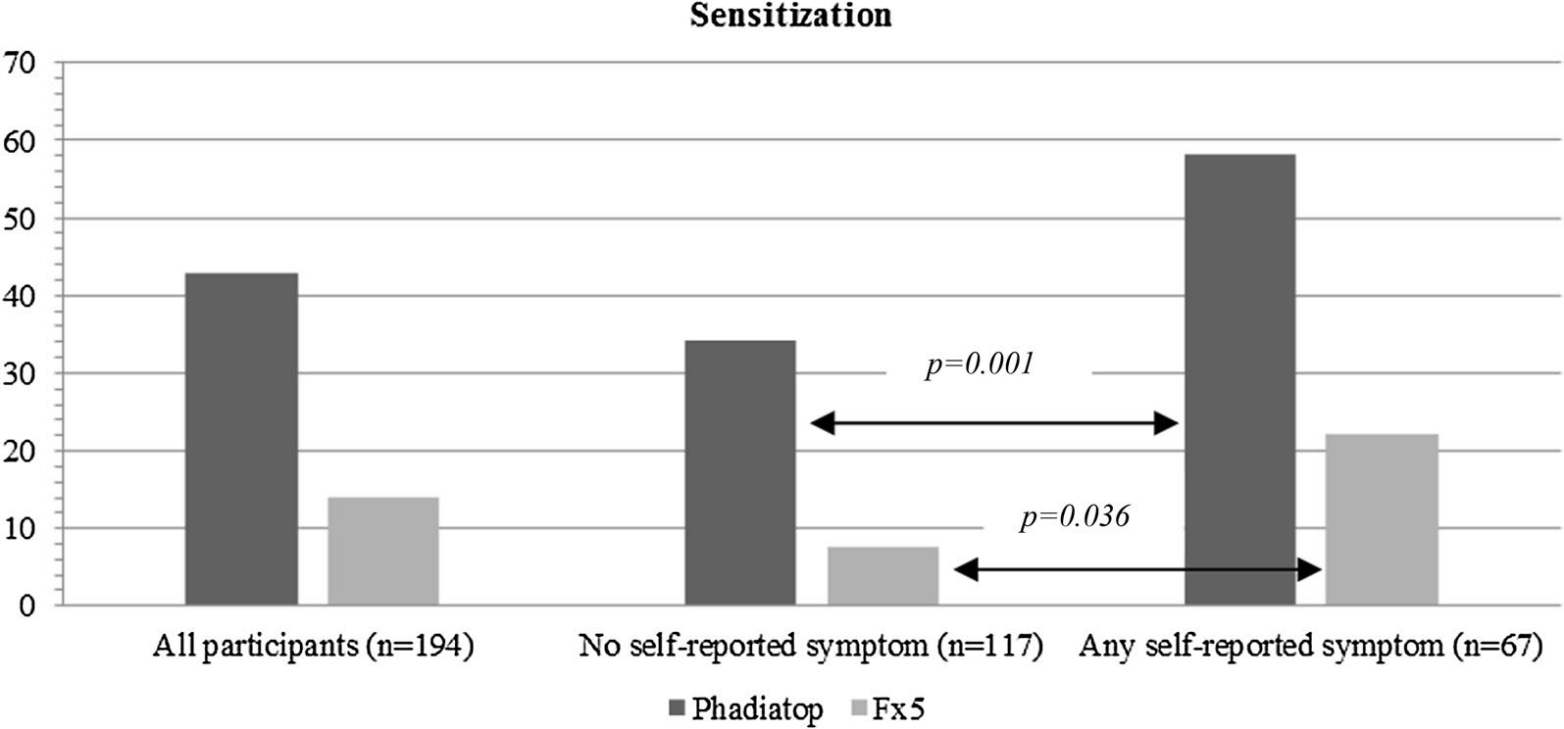
Prevalence of positive results in the airborne allergen test (Phadiatop) and the food test (fx5) for all participants with or without self-reported allergic symptom (n = 194)

According to the allergen component test (ISAC)*, 48% of the children (94/195) were positive to at least one component, while no IgE-binding was observed to any allergen component in the remaining 101 children. A positive s-IgE response to airborne allergens was seen in 43% (83/194) of the children, (Phadiatop), while a specific IgE response to the most important foodborne allergens (fx5) was only detected in 14% of the participants (27/194) (see Fig. 2).

**Fig. 3:**
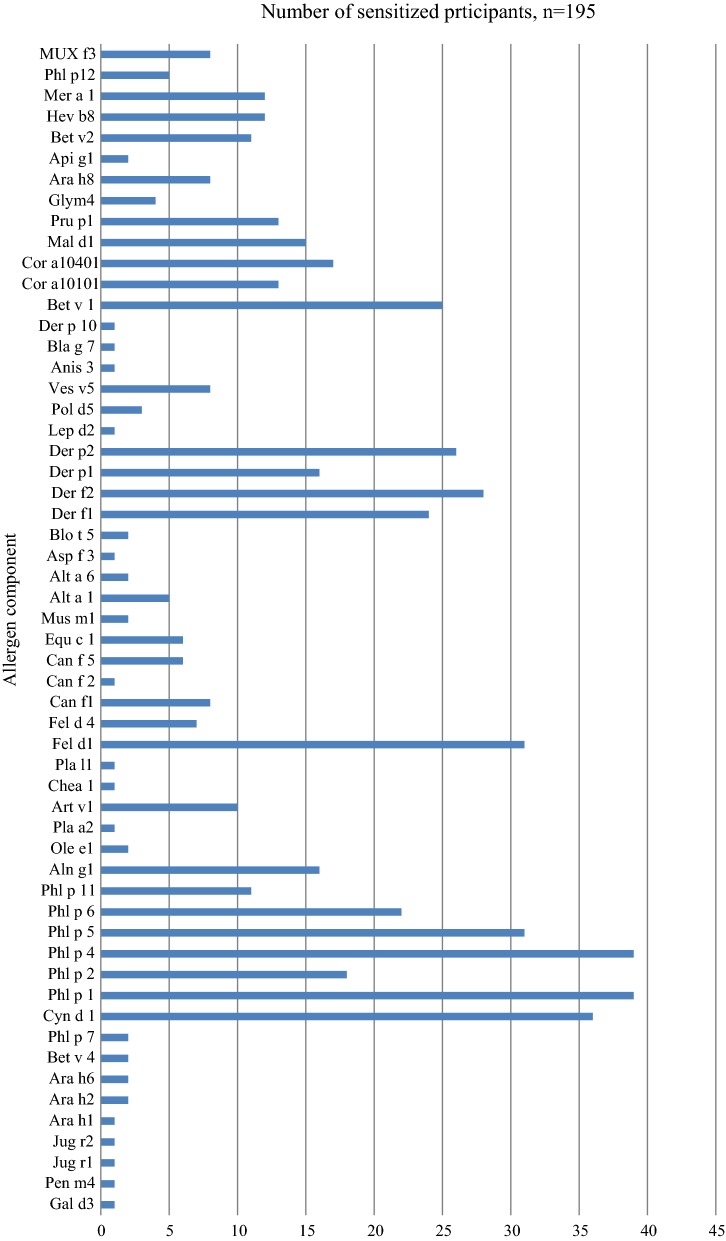
Prevalence of positive results in the ISAC test for all participants, (*n* = 195)

The findings of the ISAC test were as follows. Low sensitization to food components: none sensitized to milk, one to egg, four to nuts and seeds, two of them to peanuts (Ara h2). 31 (16%) were sensitized to birch, 23 (12%) to tree pollen (mostly to alder, 21 (11%)) and 55 (28%) to grass pollen. Sensitization to animals was found in 43 (22%), to mites in 40 (20%) and to venom in 17 (9%) (see Fig. 3). Amongst the 94 children with positive responses in the ISAC test, 40 (43%) had allergic symptom that exhibited a multi-sensitization pattern. Sensitization to venoms was found in two of the participants with eczema. The allergengroups, generally used according to the type of allergenmolecule, are explained in Table 3.

**Table 3:** Explanation of the most important allergen components in the ISAC test

Allergen component	Source	Type of allergen	Allergen group
Ara h 8	Peanut	PR-10	Food
Cor a 1	Hazelnut	PR-10	
Gly m 4	Soybean	PR-10	
Mal d 1	Apple	PR-10	
Pru p 1	Peach	PR-10	
Der f 1	*Dermatophagoides farinae*	Cysteine protease	House dust mite
Der f 2	*Dermatophagoides farinae*	NPC2 family	
Der p 1	*Dermatophagoides pteronyssinus*	Cysteine protease	
Der p 2	*Dermatophagoides pteronyssinus*	NPC2 family	
Can f 1	Dog	Lipocalin	Furry animals
Can f 2	Dog	Lipocalin	
Can f 5	Dog	Prostatic kallikrein	
Equ c 1	Horse	Lipocalin	
Fel d 1	Cat	Uteroglobin	
Fel d 4	Cat	Lipocalin	
Mus m 1	Mouse	Lipocalin	
Aln g 1	Alder	PR-10	Grass/tree pollen
Bet v 1	Birch	PR-10	
Cyn d 1	Bermuda grass	Grass group 1	
Phl p 1	Timothy-grass	Grass group 1	
Phl p 2	Timothy grass	Grass group 2	
Phl p 4	Timothy grass	Grass group 5	
Phl p 5	Timothy grass	Grass group 4	
Phl p 6	Timothy grass	Grass group 6	
Phl p 11	Timothy grass	Grass group 11	
Pol d 5	Paper wasp	Antigen 5	Venom
Ves v 5	Common wasp	Antigen 5	

**Fig. 4:**
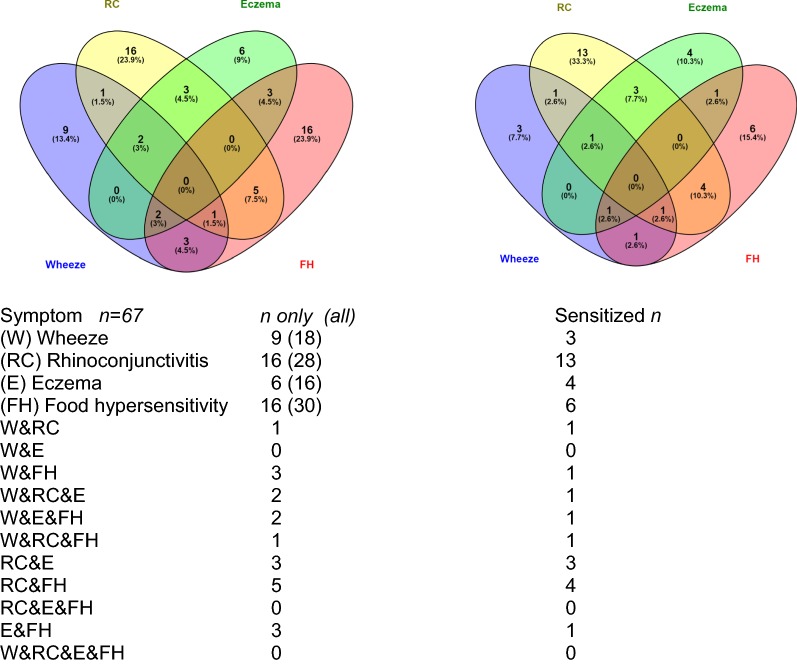
Comorbidity, assessed from the questionnaires, in relation to sensitization in the ISAC test, (*n* = 67)

Positive IgE in children with at least one allergic symptom were 58% (n = 67) compared to children without any allergic symptom 34% (n = 117) were positive in the Phadiatop. Of 28 children with rhinoconjunctivitis, 23 (85%) showed positive s-IgE results in both the Phadiatop and in the component ISAC tests. The highest prevalence of food allergens was found in the wheezing group (33%), while only 23% of the children who reported that they were allergic to certain foods had positive results. No child with eczema only showed positive s-IgE sensitization, but four of them showed positive reactions in the ISAC test. Comorbidity and sensitization are presented in Fig. 4 and Table 4.

**Table 4:** Frequencies and number of positive reactions to the allergen components in the ISAC test in children with self-reported wheeze and/or rhinoconjunctivitis and/or eczema and/or food hypersensitivity, (*n* = 67)

Allergen component	Wheeze	Rhinoconjunctivitis	Eczema	Food hypersensitivity
	*(n* = *18)* % (n)	*(n* = *28)* % (n)	*(n* = *16)* % (n)	*(n* = *30)* % (n)
Aln g1	22.2(4)	35.7 (10)	12.5 (2)	16.7 (5)
Ara h8		32.1 (9)		13.3 (4)
Bet v1	27.8 (5)	42.9 (12)	18.8 (3)	26.7 (8)
Can f1	16.7 (3)		12.5 (2)	
Can f2			12.5 (2)	
Can f5	22.2 (4)	21.4 (6)	18.8 (3)	13.3 (4)
Cor a1.0101	22.2 (4)	32.1 (9)	12.5 (2)	16.7 (5)
Cor a1.0401	22.2 (4)	35.7 (10)	12.5 (2)	20.0 (6)
Cyn d1	27.8 (5)	50.0 (14)	12.5 (2)	26.7 (8)
Der f1	27.8 (5)	25.0 (7)	43.8 (7)	20.0 (6)
Der f2	16.7 (3)	35.7 (10)	31.3 (5)	26.7 (8)
Der p1	22.2 (4)	21.4 (6)	37.5 (6)	
Der p2	16.7 (3)	35.7 (10)	31.3 (5)	20.0 (6)
Equ c1	27.8 (5)		18.8 (3)	16.7 (5)
Fel d1	33.3 (6)	42.9 (12)	31.3 (5)	26.7 (8)
Fel d4	27.8 (5)		18.8 (3)	13.3 (4)
Gly m4		21.4 (6)		
Mal d1	16.7 (3)	35.7 (10)	12.5 (2)	16.7 (5)
Mus m1	16.7 (3)			
Phl p1	33.3 (6)	53.6 (15)	18.8 (3)	30.0 (9)
Phl p2	16.7 (3)	39.3 (11)		13.3 (4)
Phl p4	16.7 (3)	46.4 (13)	12.5 (2)	20.0 (6)
Phl p5	16.7 (3)	46.4 (13)	12.5 (2)	20.0 (6)
Phl p6		39.3 (11)		16.7 (5)
Pol d5			12.5 (2)	
Pru p1		32.1 (9)		16.7 (5)

Clustering was performed on the response to allergen components and showed three broad clusters. The largest cluster was contained samples showing with no IgE or IgE to 1–5 allergen components at a low or moderate level. The two other discrete clusters contained samples with IgE reactivity to many allergen components. One of these clusters was dominated by allergen components from the grass pollen family, including Phl p 1, Cyn d 1, Phl p 5, Phl p 4, Phl p 6 and Phl p 2 in descending order of reactivity. The second cluster was dominated by moderate/high IgE responses to allergens from the mite family and the major cat allergen, including Der f 2, Der p 2, Der f 1, Der p 1 and Fel d 1 in descending order of reactivity. At least two sub-clusters were found within the pollen-dominated cluster: one in which sera reacted strongly with allergens from the mite family (Der f 2 and Der p 2), and another containing the major birch pollen allergen Bet v 1 and related allergens (Cor a 1, Aln g 1 and Mal d 1). A similar sub-cluster was found within the mite cluster, with sera also responding to Bet v 1 and related allergens (Cor a 1, Aln g 1 and Mal d 1).

## Discussion

### Principal findings

This study investigated IgE sensitization to single allergens in a population-based cohort of adolescents in the southern part of Sweden. Almost half the participants (48%) showed positive results in the ISAC test, 43% in the Phadiatop test, and 14% in the fx5 test. We found that grass sensitization was associated with rhinoconjunctivitis, and cat, mite and dog sensitization with wheeze. Two major allergen clusters were identified, which gave important information on the relevance of allergic sensitization, and the use of analytical specificity that identified the allergens/components that should be included in routine diagnostic investigations in this area, i.e. Fel d1 and Phl 1.

Our results show that wheeze is associated with sensitization to the lipocalin protein group, and that allergic rhinoconjunctivitis is associated with sensitization to the PR-10 component and *Phleum pratense* (grass pollen). The highest frequency of non-sensitization, 40%, was found among children with wheeze, which is an important finding to consider when choosing treatment for these children.

Those with food hypersensitivity formed the largest group, with no sensitization to the major allergens, but to PR-10 cross-reactive allergens. In this study, for example, only two children showed responsivity to Ara h2 (peanut) and one to walnut, but with no relation to the investigated symptoms. This in contrast to the findings of an Austrian study [4], among others, in which both walnut- and peanut-sensitized children were found. This may indicate that some of the adolsecents in the present study may have been sensitized in early childhood, but had outgrown this sensitization. It also indicates the importance of bearing cross-reactivity in mind when treating children and adolescents.

### Results in relation to previous studies

The relationship between IgE sensitization and atopic symptoms is well known from other studies all over the world, and the level of specific IgE has also been found to be positively associated with the risk of developing allergic symptoms, although other factors are also involved [5, 19–21]. Our findings regarding sensitization in relation to disease seem to be in line with the findings of Stemeseder et al. [4], who reported that 59% of the Austrian children in their cohort were sensitized. The findings that grass sensitization is associated with rhinoconjunctivitis, and cat, mite and dog sensitization to wheeze, have been described in at least one previous study [22], although the association seems to be stronger in the present study. In a Swedish population-based study from the northern part of the country, it was found that wheeze was strongly associated with specific IgE sensitization to furry animals, and was even stronger among those who had a more evolved IgE response [23]. Recently published data from the Swedish BAMSE cohort show that the presence of specific IgE is strongly associated with eczema and allergic multi-morbidity throughout childhood, and with wheeze and rhinitis from the age of 4 years, but also that over 20% of children with IgE sensitization did not develop any allergic disease in childhood. The finding that eczema was associated with sensitization to venoms was interesting, although there were only two cases in this study. This has also been reported in a population-based study by Schäfer some years ago [24]. The association between eczema and mite sensitization has also been reported in other studies, and is of great importance in the choice of treatment and wether to give information about environmental measures or not. [25].

There was a female dominance in our study that seems to be the same as in other studies, for example, the meta-analysis by Frohlich et al. [26].

Schoos et al. [27] also found a significant association between being sensitized to lipocalins and wheeze. However, their group consisted of a selected high-risk study population, whereas we studied a population-based cohort. Based on a longitudinal study, Asarnoj reported that sensitization to cat and dog allergen molecules could predict the development of symptoms of allergy to pets in adolescence [28]. Molecular-based allergy diagnostics offers new opportunities for improving the diagnosis of pet allergies [17, 28]. The results of several studies seem to be consistent in that the molecules specific for pets and pollen are useful markers for allergic wheeze and rhinoconjunctivitis, respectively. Christiansen also found similar results when studying the pattern of sensitization in children in Denmark [6].

Since allergy represents a chronic disease affecting a large number of people, the identification of valuable biomarkers is of great importance. Biomarkers could be associated with exposure, and may indicate how well the patient follows instructions to optimize medical strategies. They could also be used to evaluate tolerance development. In this study, the level of IgE was significantly higher in relation to airborne allergens than food allergens among children reporting rhinitis and eczema, but not in children reporting wheeze. Cohort studies using microarray IgE testing may provide a deeper insight into IgE sensitization profiles, and enable the monitoring of populations over time to reveal prognostic molecules before onset of the disease and this is also described in another study [20].

Investigating a well-defined age group enabled us to focus on manifestations within a certain age window. Allergic reactions to food allergens present during early childhood usually disappear before adolescence, while sensitization to environmental allergens may have already developed [29]. This was indeed seen in the present study, as the significantly higher IgE levels in response to airborne allergens than to food allergens, and also sensitization to airborne allergens in the 53 children reporting allergic symptom but less to food allergens.

Cluster analysis has been used to identify subgroups among clinical and pathophysiological diseases in other studies, and we identified two major clusters with several sub-clusters. These two major clusters are useful despite the fact that the teenagers and their IgE patterns are still developing. At least two sub-clusters were found within the pollen-dominated cluster, one in which sera reacted strongly with allergens from mites, and the other containing the major birch pollen allergen, Bet v 1, and related allergens. A similar sub-cluster was found within the major mite cluster, where sera also responded to Bet v 1 and related allergens. These sensitization profiles are very similar to those found in a Danish study by Christiansen et al. [6]. The identification of a rhinitis phenotype associated with the atopic march and investigations of risk factors in the molecular allergen pattern, will allow for the prediction of prognoses!!! and the development of personalized medicine. Adequate care of children with wheeze symptoms but no sensitization is also important.

Climate also affects allergies, as shown in a birth cohort study from the most northern part of Sweden, where children/teenagers do not become allergic to many of the allergens that are strongly correlated with wheeze in other parts of the country. This is probably due to the fact that mites cannot survive in the most northern part of Sweden due to the dry cold climate [30]. Mite sensitization is common in southern Sweden which has a wetter and warmer climate [23]. Air pollution from countries around Baltic Sea could also affect children in the general population, making them more sensitive and prone to wheeze [31, 32]. Therefore, it makes sense that sensitization to mites in this study are as common as shown among the rhinoconjunctivitis group.

### Strengths and limitations

The strengths of this study are the population-based design, and the fact that all the participants were met a doctor or a nurse. Furthermore, a dermatologist talked to all the participants during the school visit and blood test. We used the validated ISAAC questionnaire to allow comparison with the results of other investigations. We also collected information from both the participants and their parents/guardian, which further strengthens the findings. One limitation of our study is the lack of objective parameters regarding wheeze symptoms, in contrast to the symptoms of atopic dermatitis. The small study group is another limitation, although it is representative of the larger cohort.

## Conclusions

The adolescents included in this study, based on an unselected Swedish population, were mainly sensitized to grass pollen, and rarely to specific food allergens but to PR-10 components. The major grass pollen Phl p 1 was the main allergen sensitizer, followed by Cyn d 1 and Phl p 2. Sixty-one percent of those reporting any allergic symptom were sensitized, and the allergen component associated with allergic wheeze and rhinoconjunctivitis were Fel d 4, Der f 2 and Can f 5. Those with food hypersensitivity accounted for the largest group, but they were only sensitized to cross-reactive allergens.
